# Supplementary material for: Prefrontal cortical plasticity during learning of cognitive tasks
Source: Nat Commun. 2022 Jan 10;13:90. doi: 10.1038/s41467-021-27695-6 (PMC8748623; doi:10.1038/s41467-021-27695-6)
Supplement: Supplementary file 1 — Supplementary Information [file 41467_2021_27695_MOESM1_ESM.pdf]

Supplementary Information

Prefrontal cortical plasticity during learning of cognitive tasks

Hua Tang, Mitchell R. Riley, Balbir Singh, Xue-Lian Qi, David T. Blake,

and Christos Constantinidis

<u>Supplementary Fig. S1-S10</u>

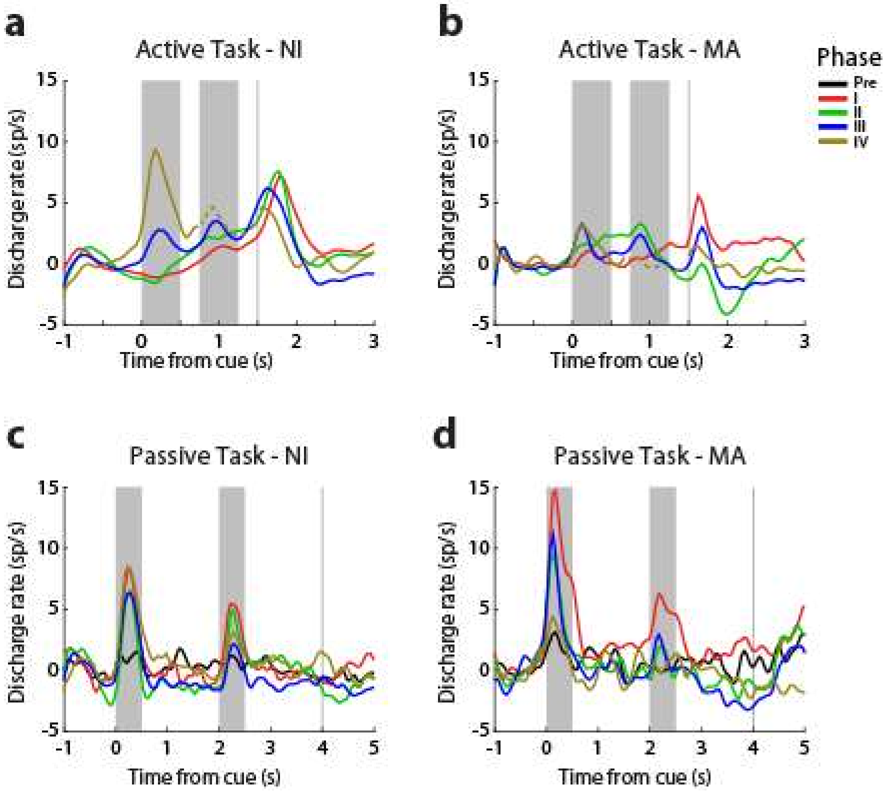

Supplementary Fig. 1. Mean firing rate of single neurons from the two monkeys. (a) Population peri-stimulus time histogram (PSTH) of responsive neurons in the active task from monkey NI (n = 515). Conventions are the same as in Fig. 1. **(c)** Population PSTH of responsive units in the passive task from monkey NI (n = 238). **(b, d)** Population PSTH from monkey MI (n = 23, and 52, respectively).

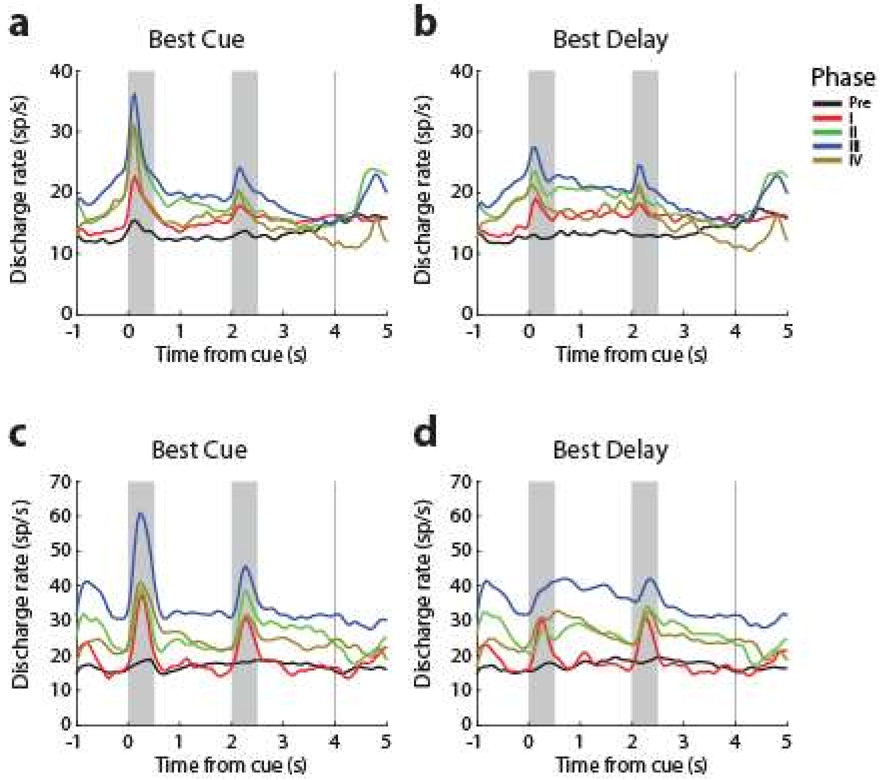

Supplementary Fig. 2. **MUA changes in activity across single channels**. **(a-b)** Population PSTH constructed based on responsive MUA units of the passive task, identified in each training phase. Data have been selected based on the presentation of the best cue (a) and delay period (b) activity, from responsive MUA units always isolated from the same electrode of subject MA (n = 324 sessions). **(c-d)** As in a-b for the second monkey subject NI (n = 213 sessions).

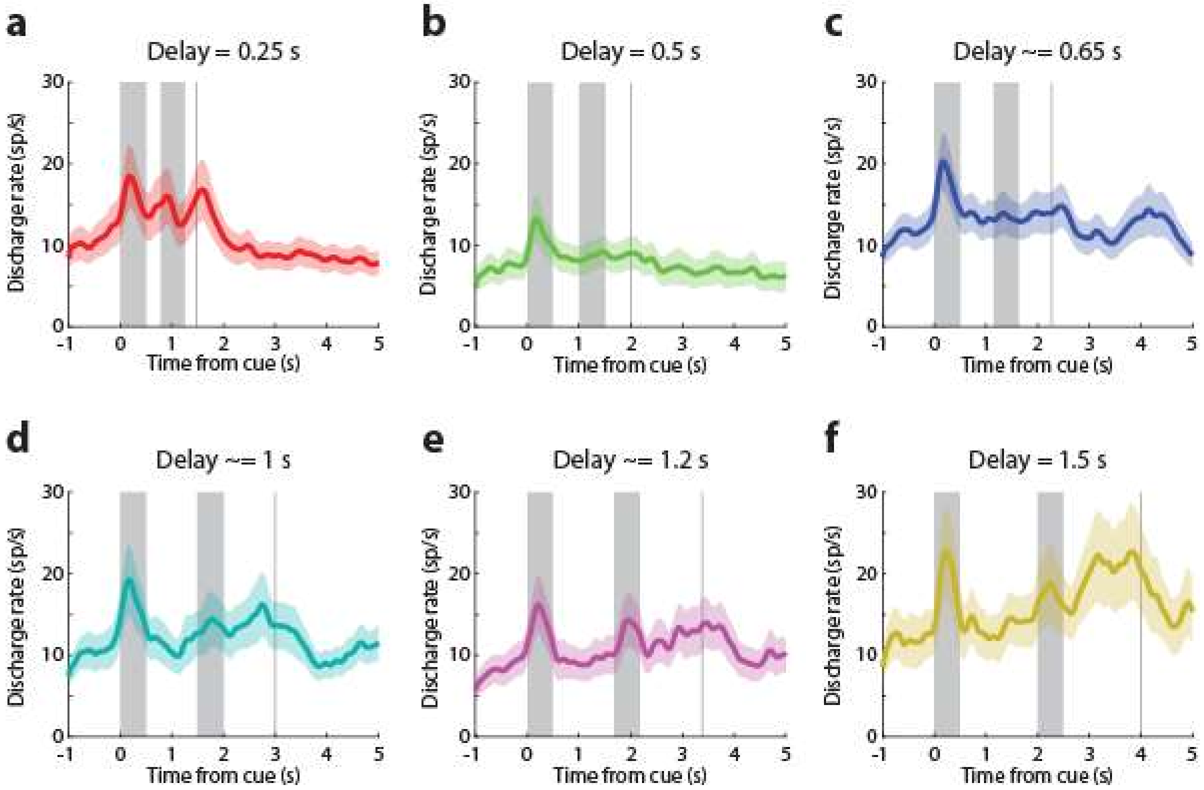

Supplementary Fig. 3. **Single neuron responses during the increase of the delay period. (a-f)** Population PSTH is drawn for the activity of responsive single neurons at different sub-phases of Phase IV of training, when the delay period of the task was progressively elongated. Details of the changing of delay length can be found in Figure 4a. Cue and sample presentations are indicated with gray bars. Shaded zones represent mean ± SEM.

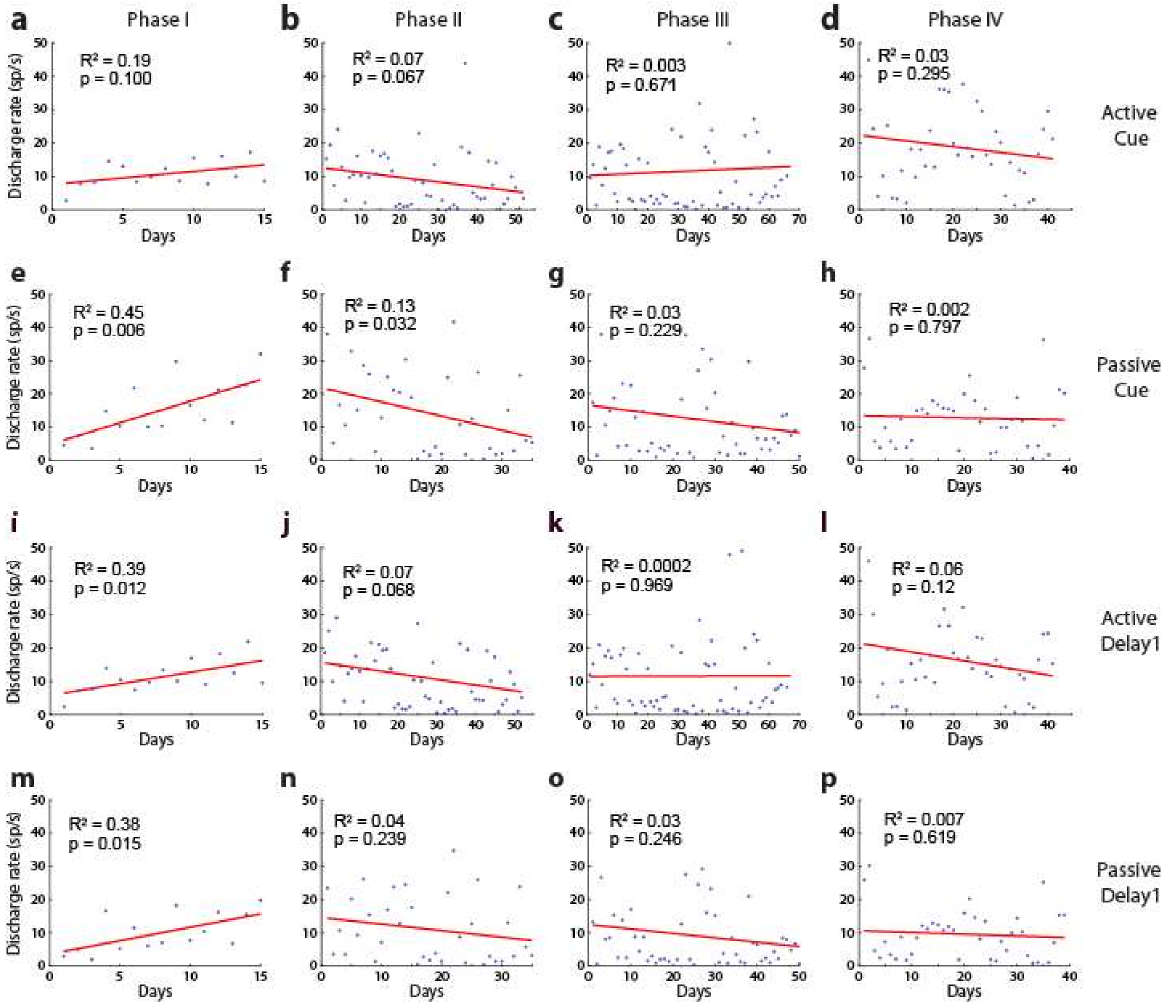

Supplementary Fig. 4. Day to Day changes of activity. Daily activity in the cue and first delay period as the active training progressed. (a-d) Activity of MUA units responsive to cues in the active task, plotted separately for each training phase. (e-h) Daily cue responses in the passive task. (i-l) Daily delay1 responses in the active task. (m-p) Daily delay1 responses in the passive task. Dots represent daily activities. Red lines represent linear regressions. R-squares indicate how much variation is explained by the regression model. The p-values indicate the significance level.

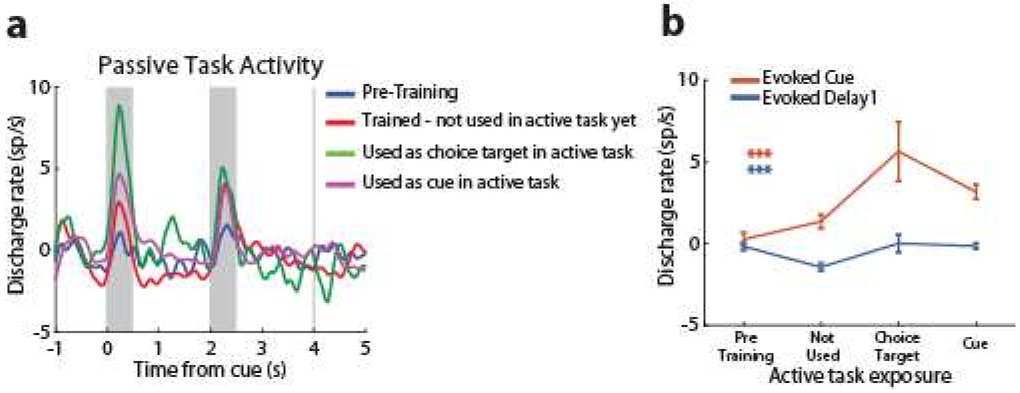

Supplementary Fig. 5. **Response to the same location across training. (a)** Mean activity of single neurons in the passive task, responding to the lower-right location, as a function of time, now grouped based on exposure to stimuli appearing in this location in the context of the active task. "Not Used" refers to passive recordings obtained at the time before the monkey had been exposed to any stimulus in this location in the active task. The "Choice Target" period begins the first time that a choice target stimulus appeared in this location, during phase III, when the cue and nonmatch stimuli appeared at diagonal locations. The "Cue" period begins the first time that a cue stimulus appeared at that location. **(b)** Mean evoked firing rate of units responded to the lower-right during the cue and delay period (n = 14/97/20/95 for each phase). Error bars represent mean ± SEM. 1-way ANOVA; $^{***}p < 0.001$.

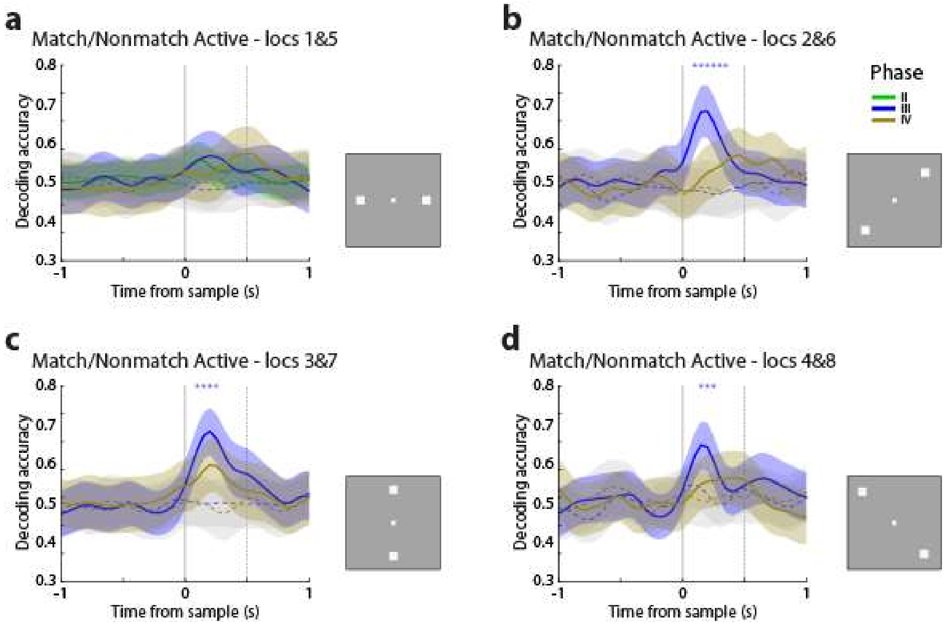

Supplementary Fig. 6. Decoding of decision information, for different location pairs. (a-d) Accuracy of decoding the match or nonmatch status of the second stimulus based on responses pooled from the active task, for match and nonmatch stimuli appearing at different location pairs (indicated by the frames on the right side) used in the task. The colored asterisks indicate a significant difference (two-sided Z-test; $p < 0.05$) between the corresponding area and the shuffled data. Dash lines represent the mean decoding accuracy of shuffled data. Shaded zones represent mean ± SD. Results were averaged from 100 resample, n = 100.

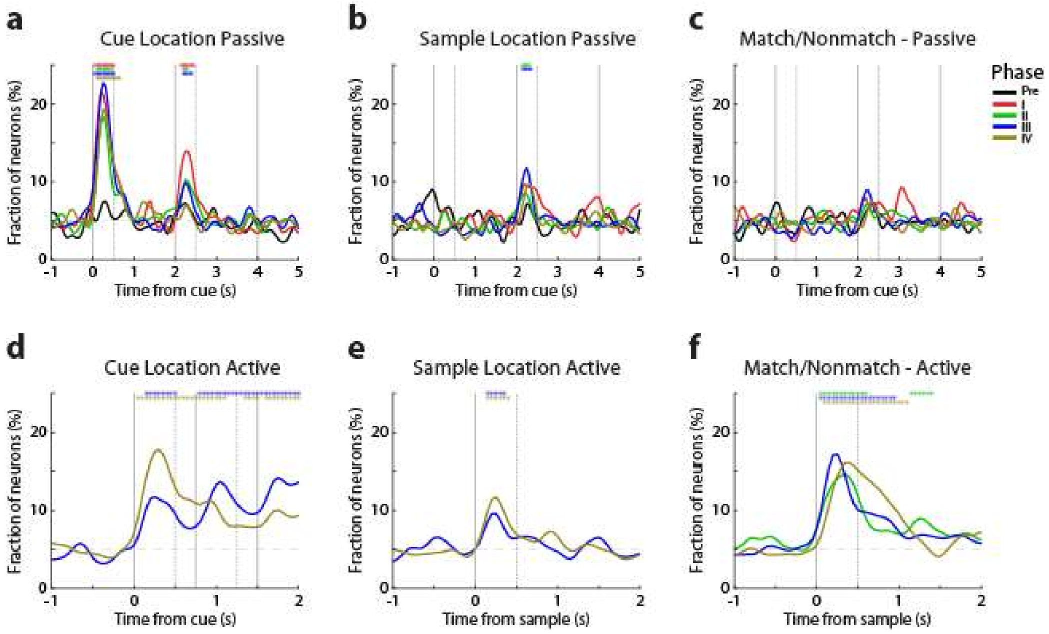

Supplementary Fig. 7. ANOVA for stimulus selectivity. a. Proportion of single neurons whose firing rate was modulated significantly by cue location in the passive task is plotted as a function of time (n = 1065). Results are shown separately from each training phase. **b**. Proportion of neurons with selectivity for the sample (second) stimulus location in the passive task at each time point. **c**. Proportion of neurons with selectivity for the match or nonmatch status of stimuli in the passive task. **d-f**. As in a-c, for the active task (n = 1093). The colored asterisks indicate time points when the proportion deviated by chance (two-sided binomial test, $p < 0.01$).

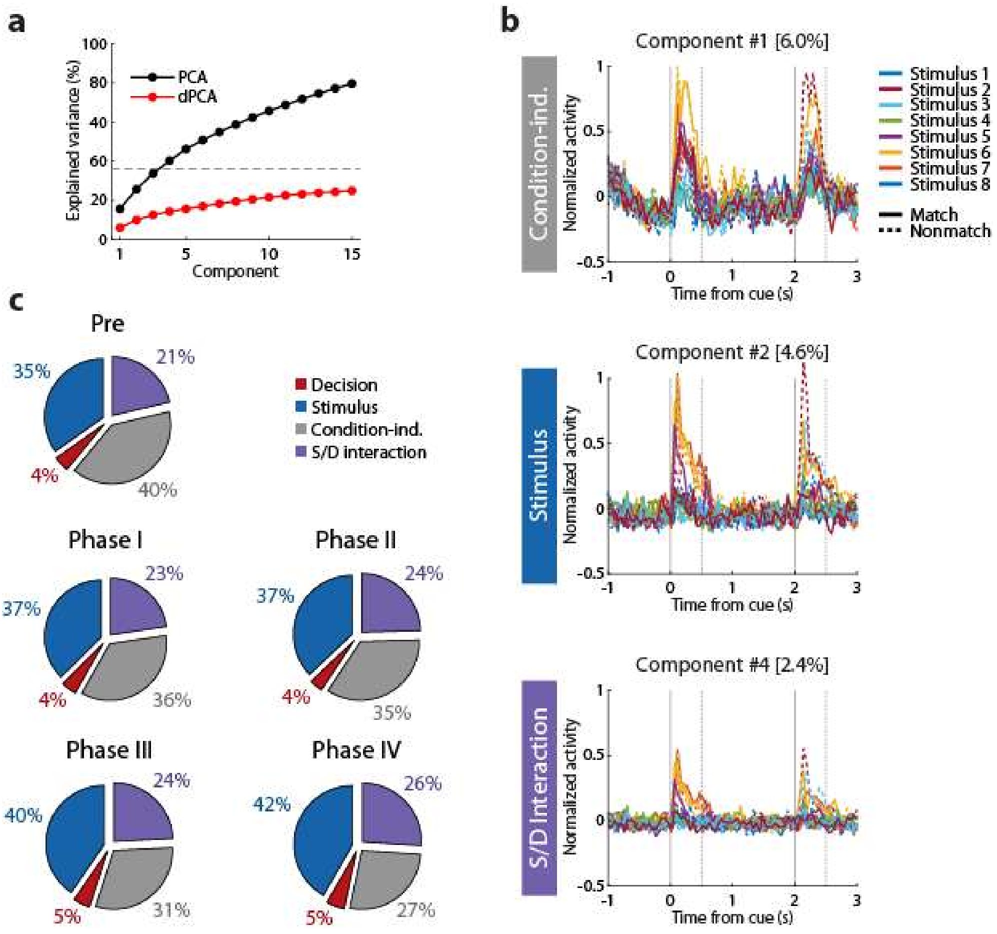

Supplementary Fig. 8. Demixed Principal Component Analysis. (a) Cumulative variance explained by PCA (black) and dPCA (red) for the passive task in Phase II. Dashed line shows an estimate of the fraction of "signal variance" in the data. **(b)** Three components of dPCA analysis based on results of Phase II: a condition independent component, a stimulus-related component, and a stimulus/decision mixture. **(c)** Pie charts represent the percentage of variance explained by each type of component (the match or nonmatch status of the trial, stimulus location, condition-independent components, and mixtures thereof) in the responses of single neurons during the passive task, across training stages.

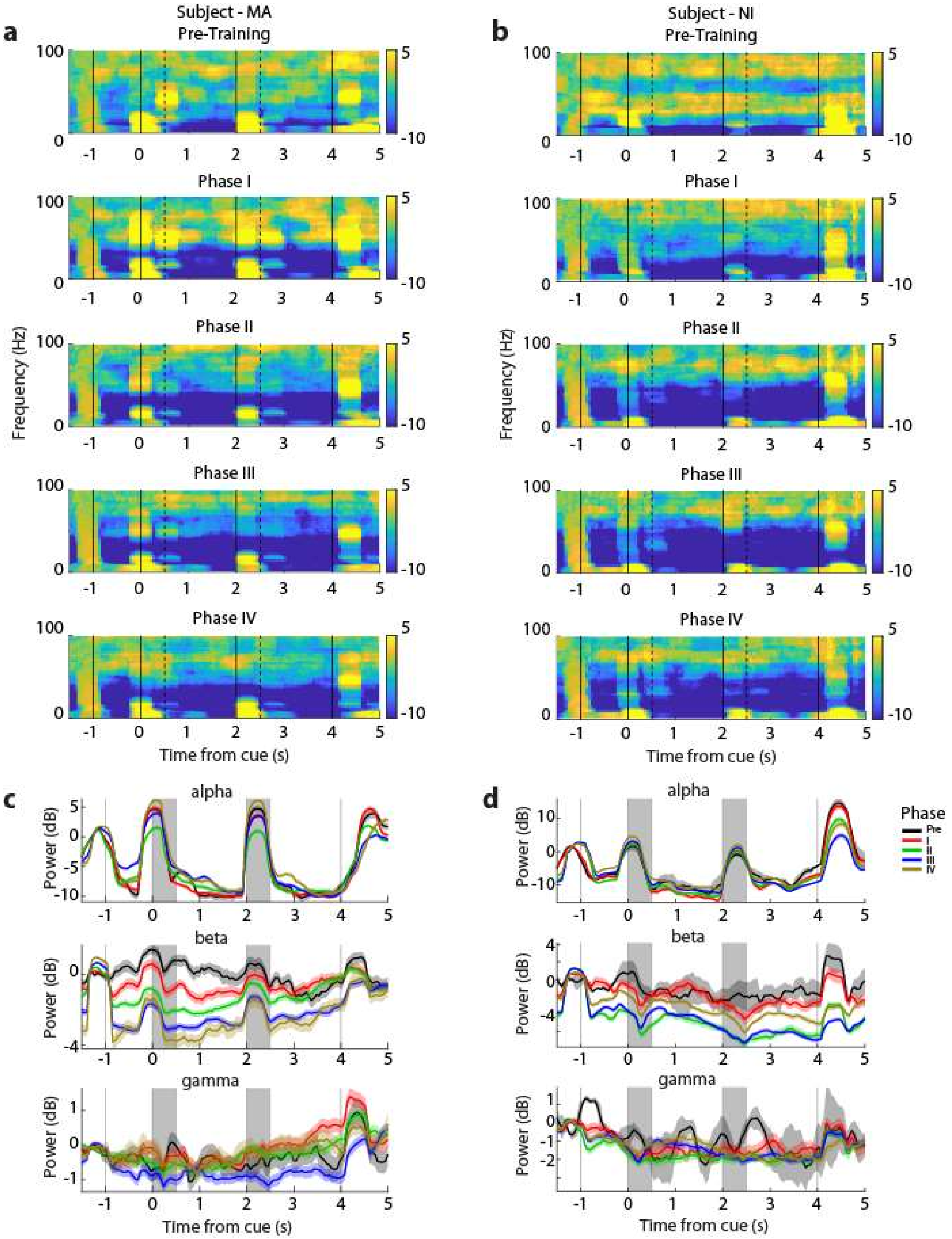

Supplementary Fig. 9. **LFP analysis in two monkeys. (a)** LFP power spectrum as a function of time for the training phases in the passive task from monkey MA. **(c)** LFP power spectrum as a function of time for the passive task, as training progressed in the active task. Time course of power at discrete frequency bands and different training phases of the active task: alpha (8-14 Hz), beta (20-45 Hz), gamma (46-70 Hz). **(b, d)** As in a, c for the second monkey subject NI. Conventions are the same as Figure 7.

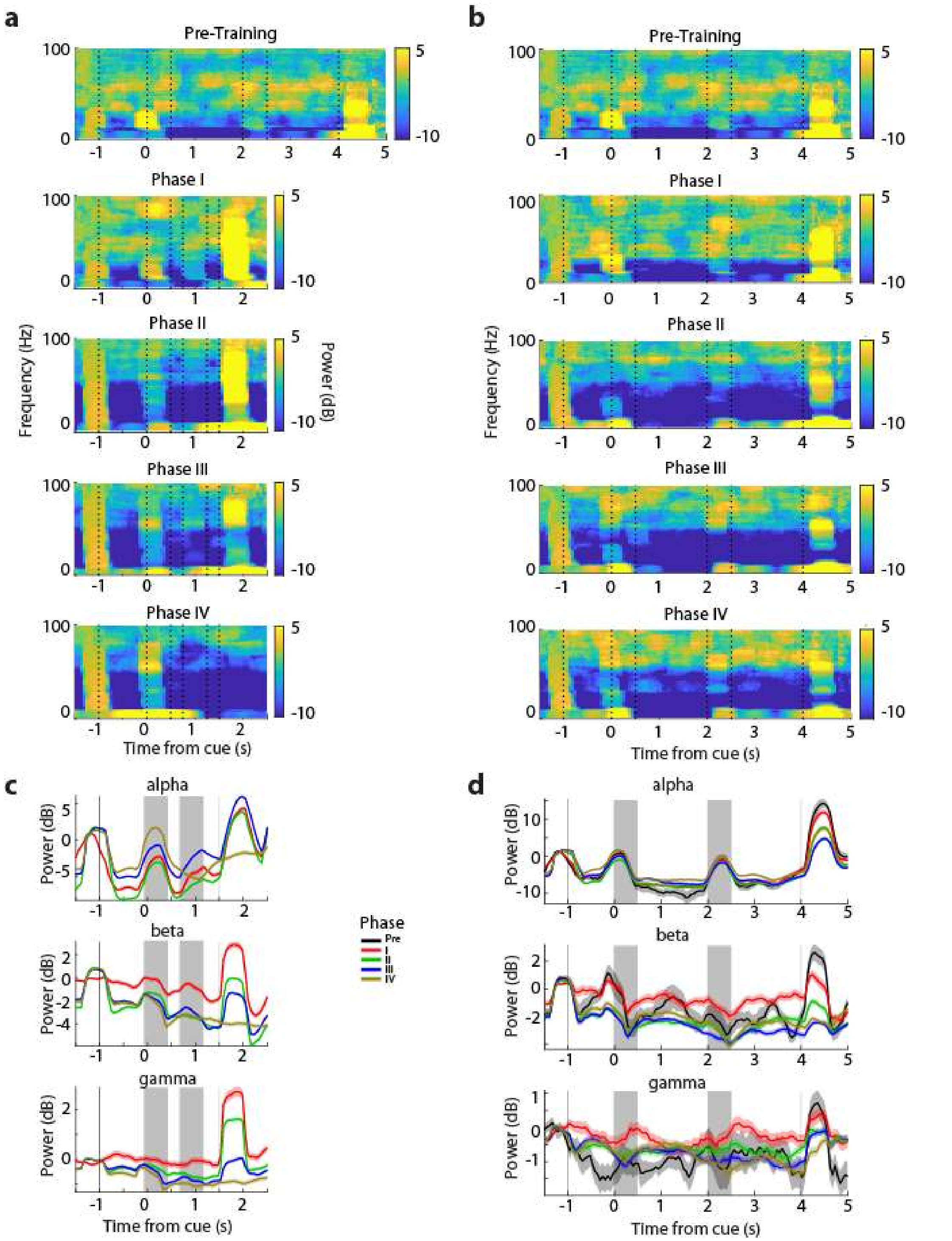

Supplementary Fig. 10. **LFP analysis in electrodes that yielded single units. (a-b)** LFP power spectrum as a function of time for the training phases in the active **(a)** and passive **(b)** tasks constructed only from electrodes that yielded single neurons (ensuring therefore that recordings were still active). **(c-d)** LFP power spectrum as a function of time for the active **(c)** and passive task **(d)**. Time course of power at discrete frequency bands and different training phases of the active task: alpha (8-14 Hz), beta (20-45 Hz), gamma (46-70 Hz). Conventions are the same as Figure 7.
